# *Leptospira interrogans* Sensu Lato in Wild Small Mammals in Three Moravian Localities of the Czech Republic

**DOI:** 10.3390/pathogens11080888

**Published:** 2022-08-08

**Authors:** Alena Žákovská, František Treml, Helena Nejezchlebová, Jiří Nepeřený, Marie Budíková, Eva Bártová

**Affiliations:** 1 Department of Biology, Faculty of Education, Masaryk University, Kamenice 753/5, 625 00 Brno, Czech Republic; 2 Department of Animal Physiology and Immunology, Institute of Experimental Biology, Faculty of Science, Masaryk University, Kamenice 753/5, 625 00 Brno, Czech Republic; 3 Department of Infectious Diseases and Microbiology, Faculty of Veterinary Medicine, University of Veterinary Sciences Brno, Palackého tř. 1946/1, 612 42 Brno, Czech Republic; 4 Bioveta, a.s., Komenského 212, 683 23 Ivanovice na Hané, Czech Republic; 5 Department of Mathematics and Statistics, Faculty of Science, Masaryk University, Kotlářská 267/2, 611 37 Brno, Czech Republic; 6 Department of Biology and Wildlife Diseases, Faculty of Veterinary Hygiene and Ecology, University of Veterinary Sciences Brno, Palackého tř. 1946/1, 612 42 Brno, Czech Republic

**Keywords:** *Apodemus flavicollis*, incidence, leptospirosis, rodents, seroprevalence

## Abstract

Leptospirosis is a widespread zoonosis, affecting humans, domestic animals and wildlife, with small mammals as a reservoir of this infection. In recent years, this disease has been re-emerging and affects approximately 1 million people all over the world each year. Due to this disease having a significant health impact, it is important to identify the source and method of infection. The risk of *Leptospira* sp. infection is higher mainly in the cities of developed and industrialised countries. The aim of the study was the detection of antibodies against *Leptospira* sp. in some wild small mammals captured in the Czech Republic. In total, samples of 855 animals captured in three locations of Moravia during a six-year study (2010–2015) were examined by a microscopic agglutination test, using eight serovars of *Leptospira interrogans* sensu lato, representing serogroups Grippotyphosa, Icterohaemorrhagiae, Australis, Canicola, Sejroe, Javanica, Pomona and Pyrogenes, as antigens. Antibodies to *Leptospira* sp. were detected in 6.1% (52/855) of animals, with a prevalence of 6.4% (51/801) and 1.9% (1/54) in rodents and insectivores, respectively. The only statistically significant difference (*p* ≤ 0.05) was in prevalence between individual species (0–33%), while there were no differences in sex (6.7% in females and 5.1% in males), locality (1.8–8%) and year of trapping (0–8.4%). Only two serovars, *L. interrogans* serovar Pomona and *L. interrogans* serovar Grippotyphosa, were detected in 5.5% and 0.5% of animals, respectively. The prevailing serovar of pathogenic *L. interrogans* s.l. can be identified in a number of infected people in the Czech Republic. The composition of vaccines should be based on the current occurrence of *Leptospira* serovars in the actual territory. For this reason, the occurrence of *Leptospira* and its serovars should therefore be regularly monitored.

## 1. Introduction

Leptospirosis is a zoonosis with worldwide distribution, caused by at least 12 pathogenic species, with more than 250 pathogens of 23 serogroups and over 200 serovars of bacteria *Leptospira* sp. [1]. The main reservoirs of this infection in natural foci are small mammals, especially rodents. Wild rats (*Rattus* spp.), especially the Norway/brown rat (*Rattus norvegicus*) and the black rat (*R. rattus*), are the most important sources of *Leptospira* spp. infection in urban and peridomestic environments, as they are abundant there. The greater prevalence of infection in rats is noticeable in geographical regions with tropical climates compared to regions with temperate climates [2]. Leptospirosis, as a health problem in developing countries, where the prevalence is more than 70%, e.g., in Brazil, Mexico, and Egypt, becomes a health problem also in developed and industrialised countries in unsanitary environments in periods, e.g., of rainfall. Humans become infected through water or aerosols contaminated with urine of infected rodents, the handling of animals, and less often by the transfusion of infected blood. Some highly pathogenic serovars of *Leptospira* cause lung haemorrhages or even lead to death. Serovars of *L. interrogans* s.l., such as Grippotyphosa, Icterohaemorrhagiae, Copenhageni, Sejroe, Bratislava, Pomona, Canicola, and Porex-jalna, have so far been isolated in the Czech Republic. The most common serovars of *Leptospira* in the Czech Republic are *L. interrogans* serovar Icterohaemorrhagiae and *L. interrogans* serovar Grippotyphosa, detected in humans [3]. The results of a longitudinal study (1993–2008) on 789 cases of human leptospirosis in the Czech Republic showed *L. grypothyphosa* (66%), *L. icterohaemorrhagiae* (21%), and *L. sejroe* (10%) as the most common etiological agents of leptospirosis [4]. In another Czech study (1994–2003) on 570 human samples, the following serovars were identified: *L. grippothyphosa* (65.6%), *L. icterohaemorrhagie* (21.1%), *L. sejroe* (10.2%), *L. bratislava* (0.9%), *L. istrica* (0.7%), *L. sorex-jalna* (0.7%), and *L. pomona* (0.4%) [5]. The occurrence of specific antibodies in wild boar (*Sus scrofa* L., 1758) correlates to a large extent with the presence of leptospires in the environment. The 100% occurrence of the *L. grippotyphosa* serotype in wild boars confirms the major occurrence of this serotype in patients in the Czech Republic [6]. *Leptospira* infections can be transmitted to humans via pets, such as dogs [4,7], but also by wild animals, such as small mammals [8], rats [2], and hares [9], and *leptospira* have also been identified in wild boars [6].

In the Czech Republic, the annual incidence of human leptospirosis does not change significantly, and a higher number of patient cases occurs with occasional outbreaks during the periodic overpopulation of wild small mammals or after unexpected flood events [9]. For example, a three-times higher incidence of leptospirosis was recorded after the floods in 1997 and 2002 [9]. With respect to the general importance of spirochaetal zoonoses and the increasing number of patients with Lyme borreliosis and leptospirosis in the Czech Republic in recent years [10], the role of reservoirs should be considered. Leptospirosis may cause very serious damage to tissues and organs and thus has a significant health impact, affecting an estimated 1.03 million humans annually worldwide and causing 58,900 deaths [11].

The aim of this study was to determine the prevalence of antibodies to *Leptospira* in some wild small mammals captured in the Czech Republic, to identify the causative agent of leptospirosis among small mammals and the serovar of *Leptospira* circulating among them.

## 2. Results

A total of 855 wild small mammals belonging to five species of rodents (*Apodemus agrarius, A. flavicollis, A. sylvaticus, Myodes glareolus*, and *Microtus arvalis*) and two species of insectivores (*Sorex araneus* and *Talpa europaea*) were trapped. Yellow-necked mouse (*A. flavicollis*) was the most frequently trapped species (58%, 497/855). The total prevalence of antibodies to *Leptospira* sp. was 6.1% (55/855). Fisher’s exact test (in the R system) was used to test the independence of leptospirosis and species because conditions of good approximation for Pearson’s chi-square test of independence were not met. For all other monitored factors, the conditions of good approximation were fulfilled. The prevalence in individual species ranged from 0% to 33%, with statistically significant differences (*p* = 0.002). The most positive species were *M. arvalis* (2/6; 33.3%), followed by *A. agrarius* (7/50; 14.0%), *A. sylvaticus* (6/57; 10.5%), *C. glareolus* (14/191; 7.3%), *A. flavicollis* (21/497; 4.2%), *S. araneus* (1/53; 1.9%), and *T. europea* (0/1). The prevalence did not differ (*p* ≥ 0.05) between sexes (6.7% in females and 5.1% in males, χ2 = 0.9277, df = 1, *p* = 0.3355), localities (1.8–8%, χ2 = 4.5436, df = 2, *p* = 0.1031), years of trapping (0–8.4%, χ2 = 9.8873, df = 5, *p* = 0.0785), and between rodents (6.4%, 51/801) and insectivores (1.9%, 1/54) (χ2 = 1.9412, df = 1, *p* = 0.1635). The 95% confidence intervals for the proportion chances for positivity could be calculated only for 2 × 2 tables, i.e., for sex and rodents versus insectivores. Females had a 1.33x higher chance of being positive than males, with a probability of 0.95 OR in the interval 0.72–2.50. Rodents had a 3.76x higher chance of being positive than insectivores, with a probability of 0.95 OR in the interval 0.62–153.83. Results, according to animal species, sex, locality, and year of trapping, are summarised in Table 1 and Table 2. Statistical differences (*p* ≤ 0.05) were found between the following species pairs: *A. agrarius* and *A. flavicollis*, *A. agrarius* and *M. arvalis*, *A. flavicollis* and *A. sylvaticus*, *A. flavicollis* and *M. arvalis*, *A. sylvaticus* and *M. glareolus*, *M. glareolus* and *M. arvalis*, *M. arvalis* and *S. Araneus*, as shown in Table 3. Only two of eight serovars of *L. interrogans* (Pomona and Grippotyphosa) were detected with 5.5% (47/855) and 0.5% (4/855) prevalence, respectively. Titres of antibodies in positive samples ranged from 200 to 3200. Samples positive for *L. interrogans* serovar Grippotyphosa had titres 200–3200, with the most frequent titre of 800 in 11 cases. Samples positive for *L. interrogans* serovar Pomona had titres 800–3200.

## 3. Discussion

Leptospirosis is an infection of global importance. Antibodies to *Leptospira* sp. were detected by MAT, e.g., in 92% of various species of rats from Philippines [12], in 68% of *R. norvegicus* from Brazil [13], and in 52% of *R. norvegicus* captured near human dwellings in Argentina [14]. Molecular methods were used in studies from the Canary Islands, where *L. interrogans* serovar Copenhageni and *L. borgpetersenii* were found in 14.8% of small mammals [15], and *L. interrogans* s.l. (*L. borgpetersenii*, *L. interrogans*, *L. kirschneri*, and *L. weilli*) was found in 7% of small mammals from Southeast Asia [16]. High prevalence of 22% was noted also in humans from Brazil, when 812 suspected cases of leptospirosis were examined from the national reference laboratory by MAT, with the most prevalent serogroup being Icterohaemorrhagie, followed by Pomona, Ballum, and Canicola [17].

In Slovakia, antibodies to *Leptospira* spp. were detected by MAT in 5% of 11 species of wild mammals [18]. In Croatia, the prevalence of *Leptospira* spp. in small rodents by MAT was 64% with serogroups *L. australis* and *L. grippotyphosa* [19] and 12.7% with serovars Sejroe, Pomona, and Australis [20]. In France, the prevalence of *Leptospira* spp. in small mammals by MAT was 30.8% with the main serovar *L. icterohaemorrhagiae* [21], 53% by MAT and PCR with the predominant serovar Icterohaemorrhagiae, followed by Sejroe, Grippotyphosa, and *L. interrogans* serogroup Australis [22], and 19.2% by RT-PCR with serotype *L. interrogans* [23]. The prevalence of *Leptospira* spp. in rats caught in six localities in Denmark in 2006–2007 was 48–89% by PCR [24]; some of the samples were examined by MAT, showing the most common serogroup to be Pomona, Sejroe, and Icterohaemorrhagiae. In rats, the prevalence of *Leptospira* spp. was recorded as 14% in England, using different diagnostic tests [25], or 45.5% in Italy, using PCR [26]. In Germany, leptospiral DNA was detected by duplex PCR in 10% of wild small mammals, with 13% in *Microtus* spp., 11% in *Apodemus* spp., and 6% in *Clethrionomys* spp. [27]. In Germany, Romanian and Slovakian harvesters working outdoors in nature had a 49% prevalence of *Leptospira* spp., as tested by MAT and confirmed by ELISA, with main serotypes being Grippotyphosa, Pomona, Bratislava, and other serogroups (Copenhageni and Pomona) [28]. New, unexpected cases of zoonoses, specifically leptospirosis, can still appear. An example is the unusual increase in icteric bovine aborted foetuses in Belgium in 2014 [29]. Most foetuses presented jaundice and splenomegaly, and cows undergoing icteric abortions had antibodies against *Leptospira* serogroups Australis or Grippotyphosa.

**Figure 1:**
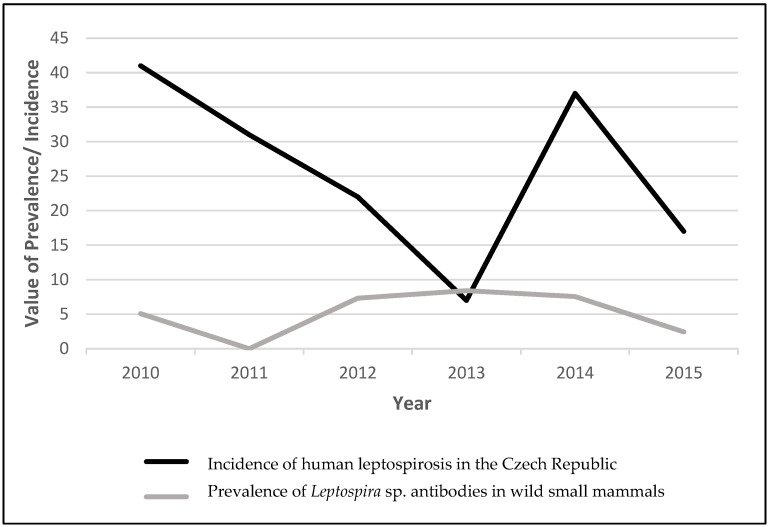
The prevalence of *Leptospira* sp. antibodies in wild small mammals compared with incidence of human leptospirosis (41, 31, 22, 7, 37, and 17 cases) in observed years 2010–2015 in the Czech Republic.

The notification rate of leptospirosis in the European Union in 2014 was 0.23 cases per 100,000 inhabitants, which represents a two-fold increase in comparison with the average number of confirmed cases in previous years. However, on the other hand, this average of the European number is equal to the average number of patients in the Czech Republic [30]. The seroprevalence of *Leptospira* in wild small mammals caught in the Czech Republic can be compared with the incidence of human leptospirosis in the observed years 2010–2015 (Figure 1, [10]), with an average of 20 human patients per year that became ill. In the following years, it was, e.g., 25 patients that suffered from disease in 2019 (approximately 0.25 cases per 100,000), 29 patients in 2020 (approximately 0.29 cases per 100,000) and 31 patients in 2021 (which corresponds to approximately 0.31 cases per 100,000 inhabitants). These data demonstrate that human leptospirosis in the Czech Republic appears annually in similar numbers [31], but occasional outbreaks are observed during the periodic overpopulation of wild small mammals or after floods, e.g., in the years 1997 (92 patients) and 2002 (94 patients). Four cases of Weil disease (leptospirosis) reported in 1997 were fatal.

However, Ref. [4] warned that the incidence of leptospirosis may be significantly underestimated, because many cases are asymptomatic or with a slight clinical manifestation and, moreover, some cases may be inaccurately diagnosed, diagnosed late, or misdiagnosed. In the Czech Republic, a decreasing trend with occasional fluctuations in the incidence of human leptospirosis was observed in 2008 compared to recent decades, but future trends are hardly predictable. Thus, the monitoring of *Leptospira* circulating in the environment is essential for a description of the actual epidemiological situation, management of the disease, and its future prevention.

Out of a total of 46 captures of small mammals from May to November, the most frequent species among 855 individuals was *A. flavicollis* (*n* = 497), followed by *M. glareolus* (*n* = 191), *A. sylvaticus* (*n* = 57), *S. araneus* (*n* = 53), *A. agrarius* (*n* = 50), *M. arvalis* (*n* = 9), and *T. europea* (*n* = 1). A similar representation of animal species was recorded in a previous study from the Czech Republic [32,33], as well as in other European countries, e.g., in Switzerland [34], Croatia [35], and Lithuania [36].

The prevalence of antibodies to eight serovars of *Leptospira* spp. was tested by MAT, which is considered to be the gold standard for the diagnosis of leptospirosis [37]. In MAT, a panel of live leptospires, belonging to recent isolates and representing the serovars circulating in the Czech Republic, were used. Individual leptospiral serovars have their typical animal reservoirs, which can be both in wild and domestic animals. The most common reservoirs are *Rattus norvegicus, Microtus arvalis*, and *Microtus agrestis* [38]. However, a large number of other vertebrates can serve as reservoirs of infection [38]. Previous studies from different regions of the Czech Republic showed a 12% [8] and 9% [39] prevalence of *Leptospira* spp. in wild small mammals, with *M. arvalis* being the most infected of five animal species. In our study, *M. arvalis* also showed the highest prevalence (33%). Among species of small mammals examined in our study, there was high variability (from 1.9% to 33.3%) in the production of antibodies against *leptospira* spp. The prevalence of *Leptospira* spp. did not differ according to the sex of animals, localities, or year of sampling. 

In the Czech Republic, serovars Grippotyphosa, Icterohaemorrhagiae, Copenhageni, Sejroe, Bratislava, Pomona, Canicola, and Sorex-jalna have so far been isolated from their main hosts, such as *M. arvalis*, *R. norvegicus*, *R. rattus*, *Mus musculus, Apodemus* sp., *S. araneus, Erinaceus* sp., *Sus* sp., and *Canis lupus* f. *familiaris* [3,31]. In our study, antibodies to two serovars of *L. interrogans* (Pomona and Grippotyphosa) were the only ones observed in a six-year study, with the range of 800–3200 and 200–3200 titres, respectively, indicating the gradual development of infection in the host organism. Serovar Grippotyphosa was also the most prevalent serovar in horses [40] and humans [41] in the Czech Republic. In contrast, serovars Copenhageni and Icterohaemorrhagiae, transmitted by rats, are usually responsible for infections in Europe [42]. This is why the geographic location, and the ecology of reservoirs, affect the prevalence of specific serovars involved in infection. Antibodies to serovar Pomona were detected only in *A. agrarius* (8.0%), which belongs to the most important host of this serovar in the Poodří locality, while antibodies against serovar Grippotyphosa were detected in six species in all three localities, which is in accordance with the results from other studies [38,43].

Based on the incidence of leptospirosis in recent years, preventive measures appear to be relatively effective. The increase in the number of patients after the floods in 1997 and 2002 is an exception. However, anti-spread measures were not effective enough during this outbreak [5]. At present, the development of a human vaccine is not an issue in the Czech Republic. However, in the field of veterinary medicine, the vaccination of domestic animals should not be neglected. The composition of vaccines should be based on the current occurrence of *Leptospira* serovars in the actual territory. For this reason, the occurrence of *Leptospira* and its serovars should be regularly monitored. Moreover, with respect to potential epidemiological risks, occasional serological surveys of reservoirs and other hosts, as well as of the local human population, should be performed.

This study presents leptospirosis as a disease that is not so epidemiologically significant in our natural conditions as it is in the tropics, but it should not be underestimated in any case. In our 6-year study, two serovars (*L. interrogans* serovar Pomona and *L. interrogans* serovar Grippotyphosa) were detected in wild small mammals. With respect to the potential risk of infection, regular monitoring should be conducted not only in humans but also in animals, especially in small mammals, as one of the main sources of *Leptospira* spp.

## 4. Material and Methods

### 4.1. Trapping of Wild Small Mammals

Small mammals were trapped using spring-loaded and live mouse traps in three Moravian localities (Poodří Protected Landscape Area, the Moravian Karst, and the Mohelno National Natural Monument) from May to November in the years 2010–2015 in a total of 46 catches (2, 4, 10, 12, 10, and 8 in individual years). The localities represented different habitat types. Poodří is situated in Northern Moravia (GPS: 49°69′98.23″ N, 18°09′00.50″ E), and trapping was carried out within the area of 10 ha in the Bažantula forest area, characterised by an oak Ficario-Ulmetum alnetosum association forest alternating with meadows. The Moravian Karst is situated in South Moravia and trapping was carried out within the area of 20 ha in the surroundings of Skalní Mlýn (GPS: 49°19′43.22″ N; 16°43′23.52″ E), which are characterised by beech forests, complemented with oak and hornbeam woods and wet meadows. Mohelno is situated in South Moravia, west of the Moravian Karst, and trapping was performed along the Oslava River in a deep canyon valley of the Mohelno Serpentine Steppe National Nature Reserve (GPS: 49°11′36.40″ N, 16°16′21.82″ E), within an area of 4 ha. Traps were placed on the ground in a line at a distance of 7 m from each other.

A total of 855 wild small mammals belonging to 5 species of rodents and 2 species of insectivores were trapped (Table 1). Animals caught by both types of traps were dissected and hearts were removed, cut, and printed on a piece of filter paper, which was placed in the fridge at a temperature of 4 °C. Blood drawn from the carotid artery of anaesthetised living individuals was used to obtain serum, which was stored at −18 °C until assays.

### 4.2. Detection of Antibodies to Leptospira by Microscopic Agglutination Test

Samples on filter papers were examined by a microscopic agglutination test (MAT) [44]. Eight serovars of *Leptospira interrogans* sensu lato (Lisl) (Grippotyphosa, Icterohaemorrhagiae, Bratislava, Canicola, Sejroe, Sorex jalna, Pomona, and Pyrogenes), belonging to serogroups Grippotyphosa, Icterohaemorrhagiae, Australis, Canicola, Sejroe, Javanica, Pomona, and Pyrogenes, respectively, represent the most prevalent *Leptospira* serovars in Europe [6]. These serovars were stored long-term in liquid nitrogen at a temperature of −196 °C. Before use, ampoules with *Leptospira* cultures were thawed and cultured at 28 °C in commercial media (Ellinghausen–McCullough–Johnson–Harris or HIMEDIA Leptospira HiVeg Medium Base, Korthof Modified, REF MV457Z, Test Line, Brno, Czech Republic), with the addition of 10% rabbit serum (Sigma Aldrich, Prague, Czech Republic), to the concentration of approximately 2 × 10^8^ leptospires per ml. The density of *Leptospira* cultures was determined using a Petroff–Hausser counting chamber after 5–7 days of cultivation. Cultures in concentrations of approximately 2 × 10^8^ leptospires per ml were used for MAT. The degree of agglutination (reaction of antigen with antibodies) was evaluated by dark-field microscopy. Samples were marked positive if more than 50% of *Leptospira* appeared to be agglutinated. Samples with titres ≥100 were considered positive.

### 4.3. Statistical Analysis

The results were statistically analysed, taking into consideration species composition, sex, locality, and year of trapping. Data analysis was performed with Pearson’s chi-square test for independence, using STATISTICA Cz 12 [45]. We tested the null hypothesis that *Leptospira* seroprevalence would not differ in species, sex, locality, and year of trapping. The differences were considered statistically significant if the *p*-value was <0.05. In the case of a statistically significant difference in seroprevalence in some of the variables, the Scheffé multiple comparison method [45] was subsequently applied. For a detailed analysis of the relationships between species pairs, the odds ratio (OR) was also calculated together with 95% confidence intervals [45].

## Figures and Tables

**Table 1:** Prevalence of antibodies against *Leptospira* spp. in wild small mammals trapped in the Czech Republic in years 2010–2015.

Years	2010	2011	2012	2013	2014	2015	Total
**Species**							
*Apodemus agrarius*			2 + 2 */14 (28.6%)	1 + 1 */18 (11.1%)		0 + 1 */18 (5.6%)	3 + 4 */50 (14%)
*Apodemus flavicollis*	3/59 (5.1%)	0/43 (0%)	7/127 (5.5%)	2/50 (4%)	8/174 (4.6%)	1/44 (2.3%)	21/497 (4.2%)
*Apodemus sylvaticus*		0/5 (0%)	1/12 (8.3%)	1/2	3/12 (25%)	1/26 (3.8%)	6/57 (10.5%)
*Myodes glareolus*		0/3	7/105 (6.7%)	1/10 (10%)	6/33 (18.2%)	0/40 (0%)	14/191 (7.3%)
*Microtus arvalis*				2/6 (33.3%)			2/6 (33.3%)
*Sorex araneus*		0/1	0/2	0/8 (0%)	0/6 (0%)	1/36 (2.8%)	1/53 (1.9%)
*Talpa europea*				0/1			0/1
**Sex**							
Female	1/32 (3.1%)	0/28 (0%)	10 + 1/137 (8%)	5/45 (11.1%)	11/111 (9.9%)	3/111 (2.7%)	30 + 1 */464 (6.7%)
Male	2/27 (7.4%)	0/24 (0%)	7 + 1 */123 (6.5%)	2 + 1 */50 (6%)	6/114 (5.3%)	0 + 1 */53 (1.9%)	17 + 3 */391 (5.1%)
**Localities**							
Mohelno			1/56 (1.8%)				1/56 (1.8%)
Moravian Karst		0/21 (0%)	8/161 (5%)	1/28 (3.6%)	14/189 (7.4%)	2/96 (2.1%)	25/485 (5.2%)
Poodří	3/59 (5.1%)	0/31 (0%)	8 + 2 */43 (23.3%)	6 + 1 */67 (10.4%)	3/36 (8.3%)	1 + 1 */78 (2.6%)	21 + 4 */314 (8%)
**Total**	3/59 (5.1%)	0/52 (0%)	17 + 2 */260 (7%)	7 + 1 */95 (8.4%)	17/225 (7.6%)	3 + 1 */164 (2.4%)	47 + 4 */855 (6%)

* Serovars of *L. grippotyphosa* and *L. pomona*.

**Table 2:** *p*-values for tests of independence of antibodies against *Leptospira* sp. of species, sex, locality, year of capture, and between rodents and insectivores.

Characteristic	Number	Positive	*p* Value
**Species**			0.002
*Apodemus agrarius*	50	7	
*Apodemus flavicollis*	497	21	
*Apodemus sylvaticus*	57	6	
*Myodes glareolus*	191	17	
*Microtus arvalis*	6	2	
*Sorex araneus*	53	1	
*Talpa europea*	1	0	
**Sex**			0.3355
Female	464	31	
Male	391	20	
**Localities**			0.1031
Mohelno	56	1	
Moravian Karst	484	25	
Poodří	314	25	
**Year of collection**			0.0785
2010	59	3	
2011	52	0	
2012	260	19	
2013	95	8	
2014	225	17	
2015	164	4	
**Rodent versus insectivore**			0.1635
Rodents	801	53	
Insectivores	54	1	
**Total**	855	54	

**Table 3:** Statistical differences in *Leptospira* sp. prevalence between wild small mammal species (OR with 95% CI for pairs of species).

Species	*Apodemus agrarius*	*Apodemus flavicollis*	*Apodemus sylvaticus*	*Myodes glareolus*	*Microtus arvalis*	*Sorex araneus*
*Apodemus agrarius*		3.69 (1.48, 9.17)	1.38 (0.43, 4.43)	2.06 (0.78, 5.41)	0.33 (0.05, 2.13)	8.47 (1.01, 71.51)
*Apodemus flavicollis*			0.38 (0.14, 0.97)	0.56 (0.28, 1.12)	0.09 (0.02, 0.51)	2.29 (0.30, 17.40)
*Apodemus sylvaticus*				1.49 (0.54, 4.07)	0.24 (0.04, 1.57)	6.12 (0.71, 52.62)
*Myodes glareolus*					0.16 (0.03, 0.94)	4.11 (0.53, 32.02)
*Microtus arvalis*						26.00 (1.91, 352.51)

## Data Availability

The data that support the findings of this study are available on request from the corresponding author. The data are not publicly available due to privacy or ethical restrictions.
